# Endogenous temporal attention in the absence of stimulus-driven cues emerges in the second year of life

**DOI:** 10.1371/journal.pone.0184698

**Published:** 2017-09-08

**Authors:** Anna Martinez-Alvarez, Ferran Pons, Ruth de Diego-Balaguer

**Affiliations:** 1 Department of Cognition, Development, and Educational Psychology, University of Barcelona, Barcelona, Spain; 2 Cognition and Brain Plasticity Unit, Bellvitge Biomedical Research Institute (IDIBELL) Barcelona, Spain; 3 Institute of Neurosciences, University of Barcelona, Barcelona, Spain; 4 Institut de Recerca Sant Joan de Déu, Barcelona, Spain; 5 Institució Catalana de Recerca i Estudis Avançats (ICREA), Barcelona, Spain; Duke University, UNITED STATES

## Abstract

Anticipating both where and when an object will appear is a critical ability for adaptation. Research in the temporal domain in adults indicate that dissociable mechanisms relate to endogenous attention driven by the properties of the stimulus themselves (e.g. rhythmic, sequential, or trajectory cues) and driven by symbolic cues. In infancy, we know that the capacity to endogenously orient attention progressively develops through infancy. However, the above-mentioned distinction has not yet been explored since previous studies involved stimulus-driven cues. The current study tested 12- and 15-month-olds in an adaptation of the anticipatory eye movement procedure to determine whether infants were able to anticipate a specific location and temporal interval predicted only by symbolic pre-cues. In the absence of stimulus-driven cues, results show that only 15-month-olds could show anticipatory behavior based on the temporal information provided by the symbolic cues. Distinguishing stimulus-driven expectations from those driven by symbolic cues allowed dissecting more clearly the developmental progression of temporal endogenous attention.

## Introduction

We are surrounded perceptually by countless different stimuli in our complex and dynamic world. Anticipating *where* and *when* an event will occur is an essential ability to optimally respond to our environment. This anticipatory behavior is closely linked to the development of attention capacities. Over the past few decades, most research has focused on spatial attention [1]. Less interest has been devoted to the temporal counterpart. Nevertheless, the ability to focus or orient attention to an instant in time is crucial, since it optimizes the deployment of attentional resources at relevant moments, overcomes the temporal limitations of working memory, and enhances perception, action, and language processing [2–4].

A well-established categorization distinguishes exogenous from endogenous orienting of attention mechanisms [5–10]. Our attention is *exogenously* oriented when it is captured by properties of the stimulus. For example, in the visual domain, a flashing light will drive our attention automatically to its location. In addition, we can also allocate our attention *endogenously* according to our current goals. For example, we endogenously orient attention towards the left side of the street when expecting our bus to come from that side.

Research in the visuospatial domain indicates that the development of exogenous and endogenous attention systems shows differing time courses during the first years of life [11,12]. During the first months, infants' attention is captured *exogenously* by certain elements in the environment (the so-called ‘obligatory attention’ period) [13]. At around 3 months of age, infants start being able to disengage their attention from its current focus to orient attention to new stimuli [12,13]. At this point, control of endogenous attention starts a slow developmental progression. The first signs of early abilities to engage endogenous attention begin to be observed at 6 months [14,15] but it is not until the end of the first year of life that a predominance of endogenous attention can be observed [16,17]. Then, an important improvement follows through the middle of the second year, and this becomes well established in toddlerhood [16,17].

In terms of the underlying mechanisms, the main characteristic of endogenous attention is that it is oriented based on internal *expectations*. These expectations can be driven by the stimulus presentation itself (stimulus-driven expectations) or can be driven by internal predictions based on previous experience/learning (goal-directed or experience-based expectations). In the first case, expectations can be derived directly from the intrinsic characteristics of the stimulation, such as the speed or direction of movement carrying spatial and temporal information. In the second scenario, predictions require a learnt association between the identity of a symbolic cue and its spatial or temporal arbitrary predictive information.

The adult literature on temporal attention has shown behavioral dissociations between these two types of expectations and also distinct brain networks involved in each of them [18–22]. On the one hand, a brain lesion or transcranial magnetic stimulation on the right prefrontal cortex alters the ability to orient attention in time based on a symbolic cue whereas effects driven by the stimuli presentation (e.g. sequential or rhythmic effects) remain spared [21,22]. On the other hand, lesions in the cerebellum affect the ability to orient attention based on stimulus-driven predictions given by the trajectory of an object [23,24]. These studies support the idea that sequential, rhythmic, or trajectory effects occur regardless of participants’ strategic allocation of attention, and hence, can be distinguished from those temporal orienting effects based on the use of symbolic cues.

Given these dissociations, distinguishing between endogenous attention driven by the stimulus characteristics and due to symbolic cues seems critical in order to have a better understanding of the developmental progression of temporal attention. In children, recent studies have started to assess children’s endogenous attention abilities distinguishing stimulus-driven effects from voluntarily or goal-directed effects due to symbolic cues revealing contradictory results. Johnson et al [18] reported that children from 6 to 16 years of age were able to orient their attention in time only in an automatic but not in a voluntarily manner (i.e. making use of symbolic temporal cues). In contrast, Mento and Tarantino [25] reported that by 6 years of age children are already able to use both. Interestingly, the endogenous effect observed did not interact with age, suggesting that by 6 years of age the ability to orient attention to a moment in time using a symbolic cue is already fully developed and comparable to that of adults. Even though this ability is present and stable at this age, it is still unknown whether at younger ages infants can build these internal expectations based on symbolic cues to endogenously orient attention in time.

Crucially for the current study, the previous studies exploring temporal endogenous attention in infancy are based on expectations driven by the characteristics of the stimuli. That is, in typical tasks assessing temporal abilities in infants, endogenous attention was assessed introducing temporal regularity, rhythmicity, or object trajectory. For example, in occlusion-based studies it was observed that 4-month-old infants perceive continuity under occlusion in low demand conditions, and by 6 months in more challenging conditions [26–28]. Similarly, early abilities to anticipate events in time have been observed at 3 months [29,30], also basic discriminative abilities between temporal intervals or the ability to detect events omitted at regular intervals at 4 months of age [29,30]. Nevertheless, all of these studies used isochronic intervals or a rhythmic regularity.

In sum, research has uncovered infants’ endogenous orienting abilities, however the distinction between endogenous attention based on *stimulus presentation* (e.g. using rhythmic, sequential, or trajectory cues) and internal predictions based on previous experience (e.g. using symbolic temporal cues) has not yet been explored.

Taken together, the reviewed studies indicate that the ability to *endogenously* orient attention in time making use of stimulus-driven temporal expectations such as rhythmicity, event regularity in blocks of identical trials, or object trajectory is present before 12 months. However, since these studies taught infants the timing by means of stimulus-driven cues, it is unknown when endogenous attention can be oriented based on symbolic cues. That is, can infants use temporal expectations if they are only based on symbolic cue information about when a target will appear? A comprehensive study of the development of endogenous attention mechanisms considering the distinction between predictions based on stimulus-driven cues and based on symbolic cues would allow a better understanding of the specific developmental progression of attention mechanisms.

Since in the spatial domain, infants can already anticipate the location and target using the predictive information of an endogenous cue by the end of the first year of life, albeit with the advantage of stimulus-driven cues [31,32], we hypothesized that endogenous temporal attention based on symbolic cues may emerge between the first and second years of life. Hence, our main goal was to study the emergence of endogenous temporal attention in infancy in the absence of stimulus-driven cues. To do so, we tested 12- and 15-month-old infants using an adaptation of the anticipatory eye movement technique (based on McMurray and Aslin (2004) [28]) controlling for (i) object trajectory, (ii) trial repetition or predictability, and (iii) temporal sequence regularity, all known to involve stimulus-driven attention mechanisms [7,20,21,22,33,34,35], and hence, the only temporal predictive information was conveyed by the symbolic cue.

## Materials and methods

### Participants

Forty-six infants from two age groups were included in the study: twenty-three 12-month-olds (M = 12.03 [range 11.25–12.17], 13 girls), and twenty-three 15-month-olds (M = 14.28 [14.16–15.19], 12 girls). They were all healthy full-term infants with no history of hearing or visual impairments. Thirty-six additional infants participated in the study but were excluded from further analysis due to fussiness, side bias, parental interference, or not completing the task. Written consent from parents was obtained before testing. The study was approved by the Bioethical Committee of the University of Barcelona.

### Procedure and apparatus

**Fig 1:**
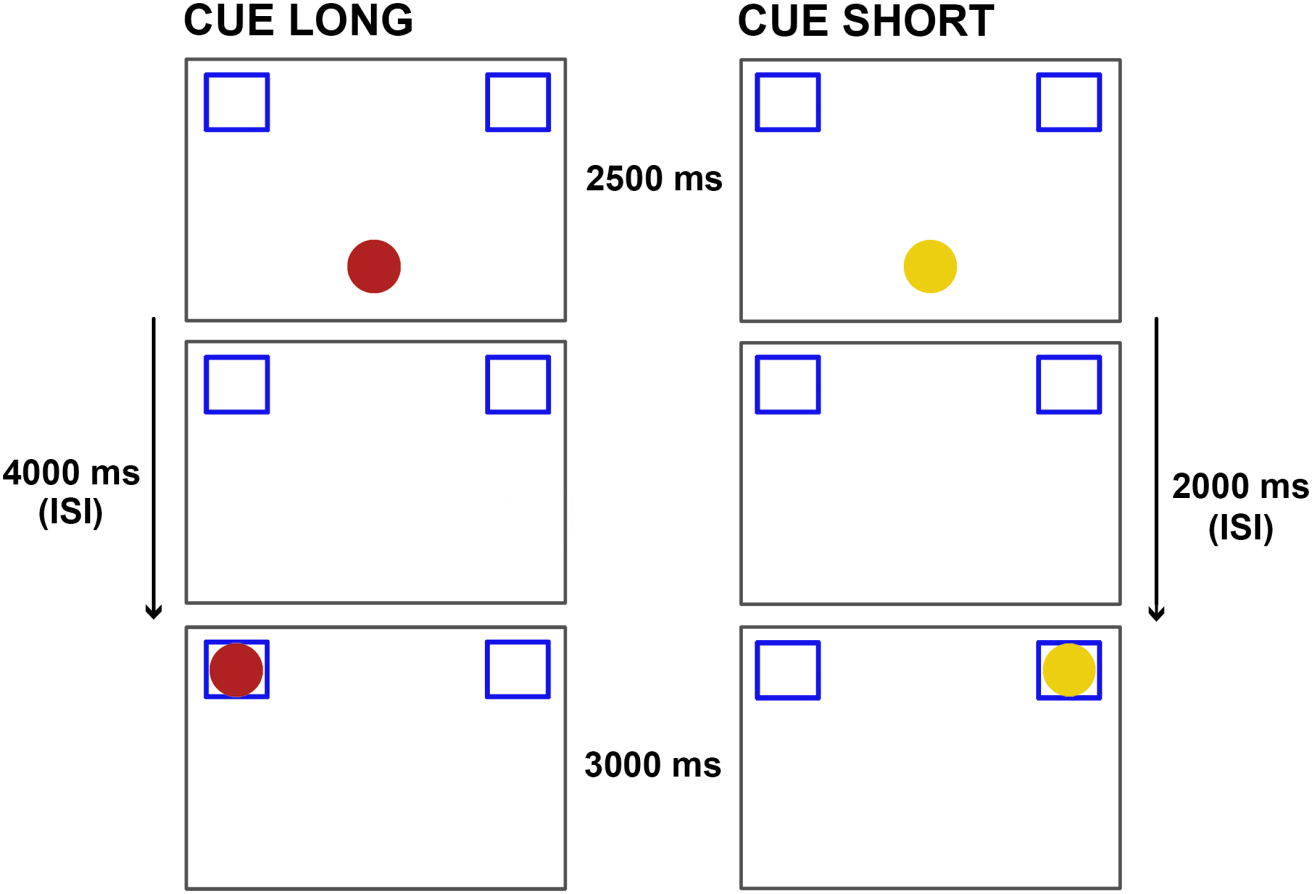
Experimental design. The cueing stimuli provided simultaneous predictive information regarding spatial location (right or left side) and temporal cue-to-target delay (short/long). Space and time was counterbalanced between participants, such that half of the participants watched the pairing left-short/right-long while the other half watched left-long / right-short. Cues were Elmo and Big Bird cartoons (here represented with a red and yellow circle respectively for illustrative purposes due to copyright restriction).

We administered 24 trials using an adaptation of the Anticipatory Eye Movement (AEM) procedure 8]. In the original AEM procedure [28] and its adaptation [31], infants were presented with a two-alternative forced-choice (2AFC) paradigm that allowed to collect repeated measures for each infant in response to each single stimulus. In both studies [28,31] each trial began with an audiovisual cueing stimulus located below a T-shape occluder. Then the cartoon moved behind the occluder at a constant velocity and then emerged from the occluder at either the left or the right side, predicted by the cueing stimulus. Once outside the occluder, the stimulus made different amusing movements and played a sound to attract infant’s attention. In our current adaptation, the occluder was removed. In each trial, the character appeared making a sound and getting bigger and smaller during 3000 ms at the bottom of the screen. It then disappeared while two empty squares at the top-left and top-right of the screen remained present during the whole trial (Fig 1). This change with respect to the previous design allowed us to avoid providing infants with stimulus-driven temporal information through the trajectory of the object. Our cueing stimuli consisted of two cartoons (Sesame Street’s cartoons Elmo and Big Bird each paired with a different sound) [31]. The audiovisual cue indicated both where (top-right square or top-left square) and when (after 2 seconds or 4 seconds) the target event was going to occur. For half the group Elmo predicted its reappearance on the right side after a long interval, and Big Bird on the left side after a short interval. For the other half, the time-space associations were reversed maintaining Elmo predicting long interval but now reappearing on the left and Big Bird a short interval but on the right (see Fig 1). Trials were presented in a semi-random fashion, with a maximum of two cuing stimuli of the same type in a row. This semi-random interleaving of short and long trials provided an additional control for stimulus-driven temporal cues such as trial repetition or predictability and allowed us to assess endogenous temporal abilities minimizing these factors. Potential sequential effects were thus kept to the minimum, with only three short trials followed by a previous short trial, and three long trials followed by a previous long trial in the whole experiment. About every four trials, an unrelated attention getter was presented to re-engage infant attention [28,31].

Testing took place in a dimly lit, sound-attenuated laboratory room. During the experiment, infants were seated in an infant seat approximately 60 cm in front of a 17-inch TFT monitor with a display resolution of 1024 x 768 pixels. Two stereo loudspeakers played the stimuli at 65 ± 5dB. Stimuli were presented on the computer monitor using Tobii Studio software and a Tobii X120 standalone eye tracker recorded eye movements at a sampling rate of 60 Hz. The Tobii eye tracker’s five-point calibration routine was used. Once the calibration routine was successfully completed, the experiment started.

### Data analysis

For the temporal and spatial analyses, the 24 trials were divided into three blocks. The screen was divided in three equal areas (left, center, and right), and two areas of interest (AOI) were used for the analysis: left and right [31]. In order to analyze the data, at least one anticipatory look for each cue type in each block was necessary. Data from six infants were excluded because they did not reach this criterion.

The spatial analysis assessed whether infants learned the location of the target reappearance. For each trial and infant, the percentage of total looking time (PTLT) to each AOI (left or right) between the disappearance of the cue and reappearance of the target (i.e. the full available time interval per trial) was calculated [31].

The temporal analysis assessed whether infants adapted their anticipatory looking behavior according to different cue-to-target delays (short or long) predicted by the cue. For this analysis, the time window shared by the two cue types, that is, the 2-seconds period after the cue disappearance, was established as the anticipatory period [31]. The latency of the first saccade to one of the two AOIs during the anticipatory period was obtained for each trial.

In addition, despite we tried to minimize as much as possible sequential effects and given that these effects appear even after two consecutive trials we also analyzed potential sequential effects in the experiment. To do so, mean latencies were calculated for each participant and trial type: a) short delay preceding a current short; b) long delay preceding a current short; c) short delay preceding a current long; and d) long delay preceding a current long. Therefore, (a) and (b) indexing sequential effects at the short interval, whereas (c) and (d) index sequential effects at the long interval. One participant did not have at least one data point for one of the trial types at short interval and thus was not included in the short interval analysis.

The previous analyses are based on a non-a priori assumption of one domain (spatial/temporal) depending on the other, by examining infants’ spatial anticipation irrespective of the temporal information and temporal anticipation irrespective of spatial information. In a final analysis, we assessed whether infants were able to anticipate both at the expected location and expected time (the spatio-temporal anticipation analysis). To do that, we combined measures of spatial anticipation (PTLT to the correct side) and temporal anticipation (analysis restricted to the time-window just before the onset of the target reappearance predicted by the cue (500 ms before reappearance).

## Results

### Spatial anticipation

**Fig 2:**
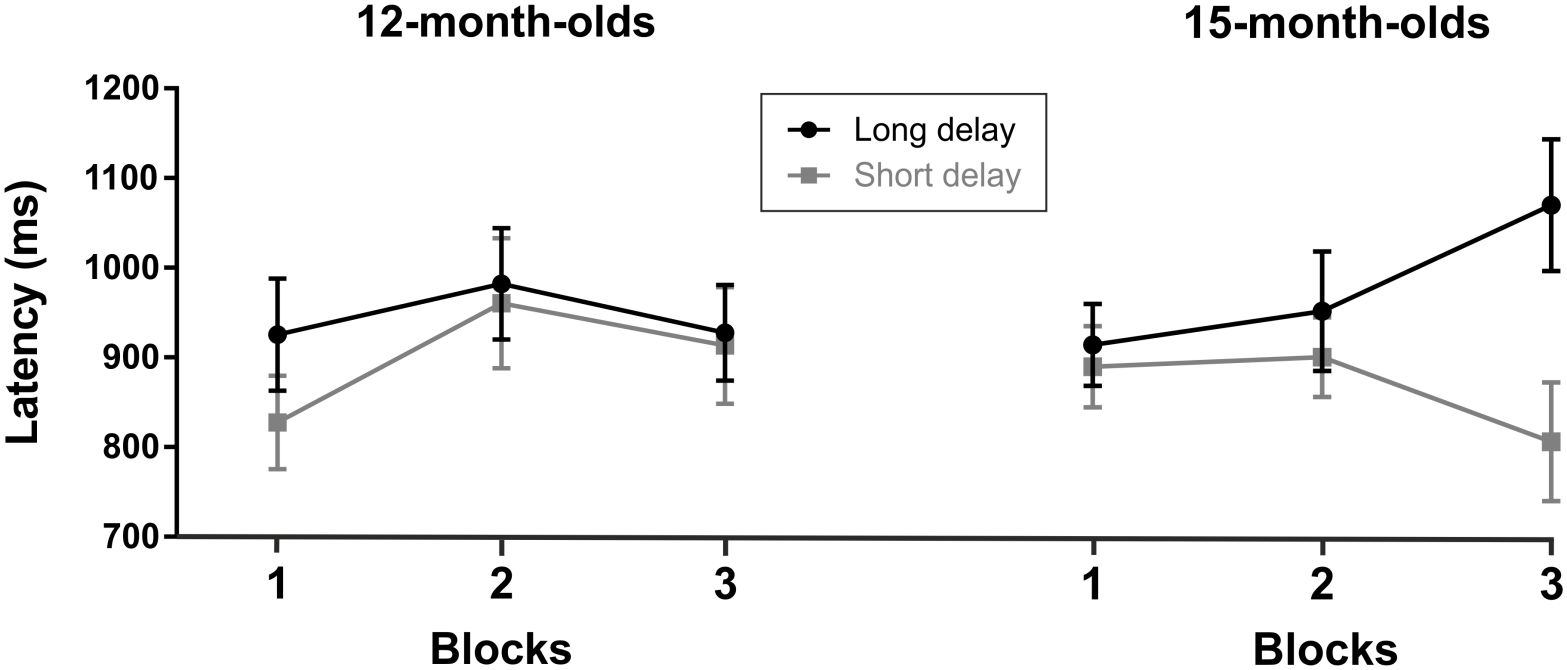
Percentage of total looking time (PTLT) at correct side during the inter-stimulus-interval (ISI) for each age group in each of the three blocks. Bars indicate standard error of the mean.

We submitted the PTLT scores to a repeated-measures analysis of variance (ANOVA) with block (first vs. second vs. third) as the within-subjects factor, and age (12 or 15 months) as the between-subjects factor. Only an effect of block was observed [*F*(1, 44) = 8.886, *p* = .005; η^2^ = .177] with no significant interaction. *T*-test comparisons of means against chance revealed significant differences in the third [*t*(45) = 2.410, *p* = .020], but not in the first [*t*(45) = 1.572, *p* = .123], or second block [*t*(45) = .380, *p* = .706]. These results indicate that both groups of infants learned to anticipate the location of the target (see Fig 2). This analysis provided evidence of the reliability of our AEM adaptation, with both groups of infants showing significant spatial learning effects.

### Temporal anticipation

We analyzed the latency measures with a mixed repeated-measures analysis of variance (ANOVA) with block (first vs. second vs. third) and length of delay (short vs. long) as within-subject factors, and age (12 or 15 months) as the between-subjects factor. The ANOVA revealed a significant effect of delay [*F*(1,44) = 7.282, *p* = .010; η^2^ = .142], and a significant block x delay x age interaction, [*F*(1,44) = 4.752, *p* = .035; η^2^ = .097].

**Fig 3:**
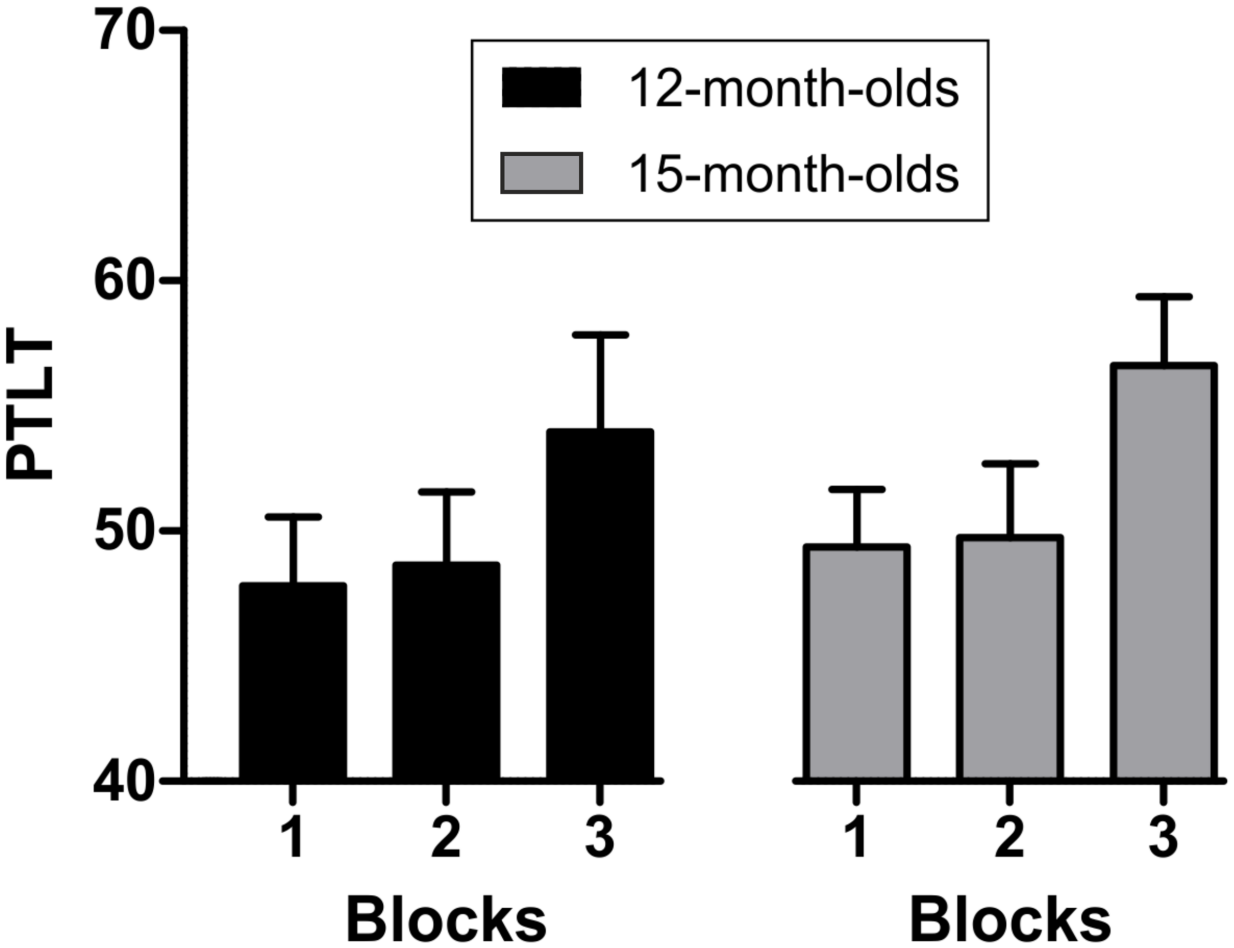
Mean latency (in ms) of first anticipatory look for *short* and *long* delays in the first two seconds after the cue in each of the three blocks for each age group. Bars indicate standard error of the mean.

To determine the source of the interaction we conducted planned comparison analyses of the latency measures at each age. For the 12-month-old group no significant effect of delay, [*F*(1,22) = 1.194, *p* = .286], neither block x delay interaction, [*F*(1,22) = .829, *p* = .373] were observed. For the 15-month-old group, a significant effect of delay [*F*(1,22) = 7.431, *p* = .012; η^2^ = .252], and a significant block x delay interaction [*F*(1,22) = 4.290, *p* = .050; η^2^ = .163] were observed. A follow-up analysis of this interaction revealed a significant difference in the latency between the *short* (M = 805 ms, SD = 310ms) and the *long delay* (M = 1062 ms, SD = 344 ms) with longer latencies for the long than the short delay only in the third block (*t*(22) = 2.756, *p* = .012; in the first two blocks *ps* > .1 (See Fig 3). These results indicate that 15-month-olds, but not 12-month-olds learned to anticipate according to the temporal cue-to-target delay predicted by the cue.

Additional analyses were conducted to evaluate potential sequential effects within the cohort. The ANOVA with current trial (long/short) and previous trial (long/short) revealed no significant effect of the previous trial [*F*(1,40) = 0.553, *p* = .461] neither an interaction [*F*(1,40) = 0.206, *p* = .652]. These results indicate that, with the current design, the length of the previous trial interval does not influence infants’ behavior.

### Spatio-temporal anticipation

The spatio-temporal analysis investigated infants’ anticipatory abilities in both spatial and temporal domains at the same time. We analyzed their looking behavior at the expected location around the expected time window of the relevant event (500 ms prior to target reappearance). As the previous analysis revealed that only 15-month-olds -but not 12-month-olds- adapted their behavior according to the temporal information, we expected that only the oldest group would look longer to the correct side at the expected time.

**Fig 4:**
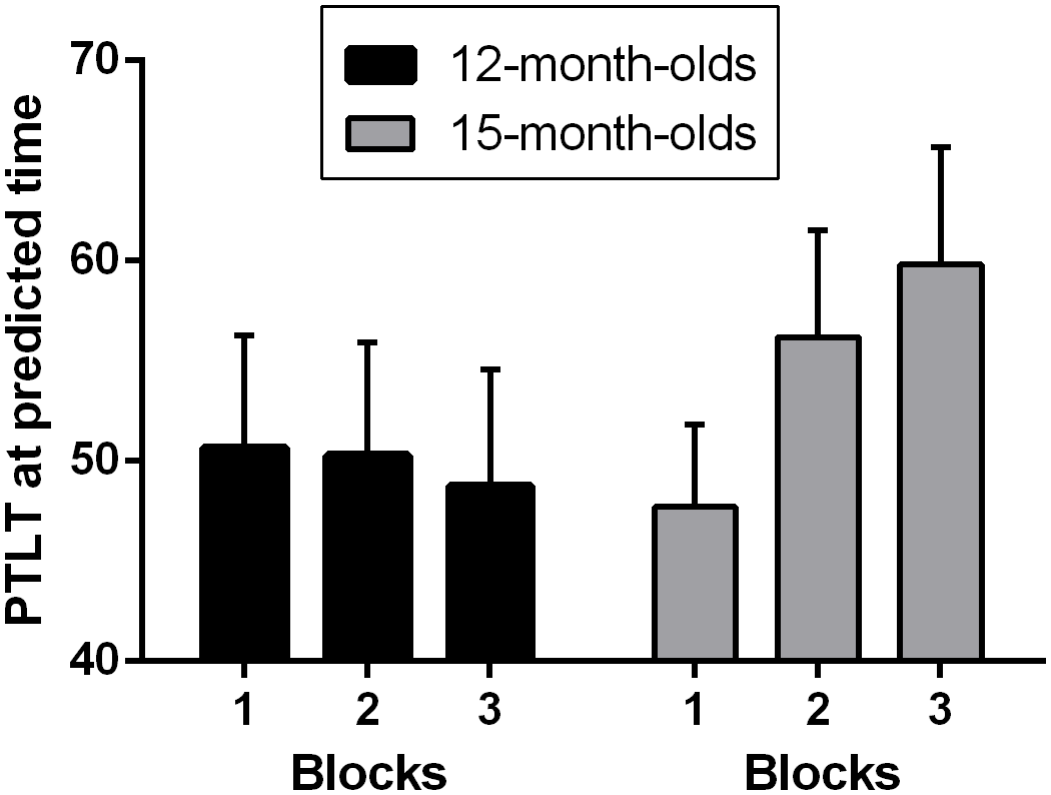
Percentage of total looking time (PTLT) at correct side during the expected time (500ms prior to target) for each age group in each of the three blocks. Bars indicate standard error of the mean.

For each age group, we submitted PTLT scores during the 500 ms prior to target to a one-way ANOVA with block as a factor (first vs. second vs. third). As expected, while no significant block effect was observed in the younger group of infants [*F*(1,22) = 0.048, *p* = .829], a significant block effect was observed in the older group [*F*(1,22) = 4.638, *p* = .042; η^2^ = .174] (see Fig 4). For the 15-month-old group, the analyses confirmed a linear learning effect, with a significant difference between the first (M_PTLT_ = 48, SD = 20) and last block (M_PTLT_ = 60, SD = 28) (*t*(22) = 2.15, *p* < .05). These results provide further evidence that 15-month-olds, but not 12-month-olds, seem to be able to learn to adapt their anticipatory behavior according to both spatial and temporal predictive information.

## Discussion

Previous infant research investigated endogenous attention abilities. However, in terms of the underlying mechanisms of this cognitive ability, the distinction dissociating endogenous attention based on stimulus presentation (e.g. using rhythmic, sequential, or trajectory cues) from endogenous attention based on previous experience (e.g. using symbolic temporal cues) was not investigated yet. By distinguishing between stimulus-driven and goal-directed/experience-based expectations we have been able to dissect more clearly the developmental progression of temporal endogenous attention. We eliminated the stimulus-driven cues introduced in previous infant methodologies and built an experimental task that controlled for the influence of these effects in order to investigate endogenous temporal attention driven by symbolic cues. Based on existing data on the ability to make endogenously driven predictions in the visuo-spatial domain, we hypothesized that endogenous temporal attention should begin emerging between the first and second years of life. Our findings confirmed our prediction by showing that, in the absence of stimulus-driven cues, 15- but not 12-month-olds are able to endogenously orient attention in time.

Orienting of attention depends upon expectations established either by *exogenous* mechanisms, already available during the first months of age, or *endogenous* mechanisms that develop later in life [11,17,29]. Recent adult evidence has revealed that in the temporal domain these endogenous expectations show distinct behavioral effects and brain when these are driven by to the properties of the stimulus themselves and from those driven by symbolic cues [21,22]. In our task, endogenous orienting of attention was explored controlling for the presence of stimulus-driven cues that could guide infants' attention more automatically. Prior to target appearance, informative cues manipulated their expectations about *where* and *when* the upcoming target would appear by predicting spatial location and cue-to-target temporal interval. Therefore, the current adaptation of the AEM method [28] enabled us to examine both spatial and temporal endogenous orienting mechanisms within the same experimental design and with the same set of participants. With this experimental design, our results show that although both 12- and 15-month-olds could predict on which side of the screen a cue would reappear, only 15-month-olds adapted their anticipatory behavior according to the predicted temporal information.

Importantly, the current AEM procedure adaptation allowed us to have a better understanding of infants’ endogenous abilities driven by symbolic cues. First, as there was no occluder, infants could not use the predictive information provided by movement trajectory. Hence, stimulus-driven cues driven by object trajectory tracking were not available to infants as in previous studies [28,31]. Second, the trial-by-trial cue appearance was pseudo-randomized with no more than two consecutive trials of the same type appearing in a row. That is, we minimized potential sequential effects (another source of stimulus-driven influence) that may obscure endogenous orienting abilities based on symbolic cues. The absence of sequential effects in our study may be related to methodological factors. Due to the fact that reaction time (RT) is the measure that has been previously found to indicate sequential effects [19,36–39], one possible source of divergence relates to the method used in the current developmental study. Given that this is, as far as we know, the first study on endogenous temporal attention in infants, it is possible that the eye tracking methodology used may show a different sensitivity compared to behavioral methodology used to assess sequential effects with children and adults [18,19,25,35–39]. However, another explanation would relate to the low number of trials in some of the conditions included in this analysis. That is, with a maximum of 3 trials of short trial previous short interval in the whole experiment. Therefore, our experimental design -used to investigate purely endogenous temporal attention (i.e. trying to minimize sequential effects)—could have added high variability/noise in the data contained within the analysis of sequential effects. Further infant studies directly addressing sequential effects are needed to investigate this aspect further. Finally, due to the fact that instead of using blocks with the same duration, trials randomly alternated the cue-to-target delay (*short*, 2 seconds, or *long*, 4 seconds), temporal sequence regularity could not help infants learn the contingency. The challenging conditions of the current AEM adaptation may have caused the spatial and temporal orienting effects observed only in the last block of the experiment. In contrast, with tasks involving stimulus-driven cues one would expect learning of endogenous information to be boosted and arise after just a few trials [26].

In terms of temporal attention development, the emergence of infants’ endogenous attention in the second year of life is in line with Mento and Tarantino [25] results, which demonstrated that cognitive mechanisms underlying endogenous temporal predictions by symbolic cues are fully established at six years of age. Therefore, our results provide an important piece of evidence for the developmental literature by showing that this ability seems to emerge by the age of 15 months.

Regarding the spatial predictability factor on temporal orienting of attention, the current results extend previous research in children and adults [18,25,40,41] by showing that when the cues are both spatial and temporally predictable, infants at 15 months of age, but not earlier, are able to use temporal expectations to adapt their behavior.

Finally, from a general cognitive perspective, it is important to notice the adaptive value of the developmental trajectory observed for temporal attention with other domains with intrinsic temporal characteristics, such as language. The early available temporal orienting of attention is reflected in infants’ early sensitivity to perceiving the rhythmic characteristics of language at birth in discriminating the stress patterns in different languages [42]. On the other hand, a more elaborated endogenous control of attention emerges at the second year of life, the moment when the ability to extract hierarchical structures that rely on dependencies between temporally distant relationships (e.g., **is** play**ing**, **is** cry**ing**) is observed [43–45]. This type of learning requires focusing attention on relevant elements in speech carrying the rule while disregarding irrelevant information [43]. Therefore, an important question for future research is how these two types of endogenous abilities to orient attention in time may assist or boost other aspects of cognitive development that involve temporal processing, such as language [46]. In fact, recent studies have provided evidence supporting the idea that selective attention to specific moments in time while perceiving speech predicts language outcome in preschool children [47]. In a similar vein, attention modulations given by the characteristics of the speech input have an effect on how well language rules are learned in infancy [43,44,48, 49]. It may thus be worth exploring further how the emergence of the ability to orient attention in time endogenously may serve as a scaffold for language development [50].
