# Correction: Endogenous temporal attention in the absence of stimulus-driven cues emerges in the second year of life

**DOI:** 10.1371/journal.pone.0190734

**Published:** 2018-01-02

**Authors:** Anna Martinez-Alvarez, Ferran Pons, Ruth de Diego-Balaguer

The images for Figs 2 and 3 are incorrectly switched. The image that appears as Fig 2 should be Fig 3, and the image that appears as Fig 3 should be Fig 2. The figure captions appear in the correct order.

**Fig 2:**
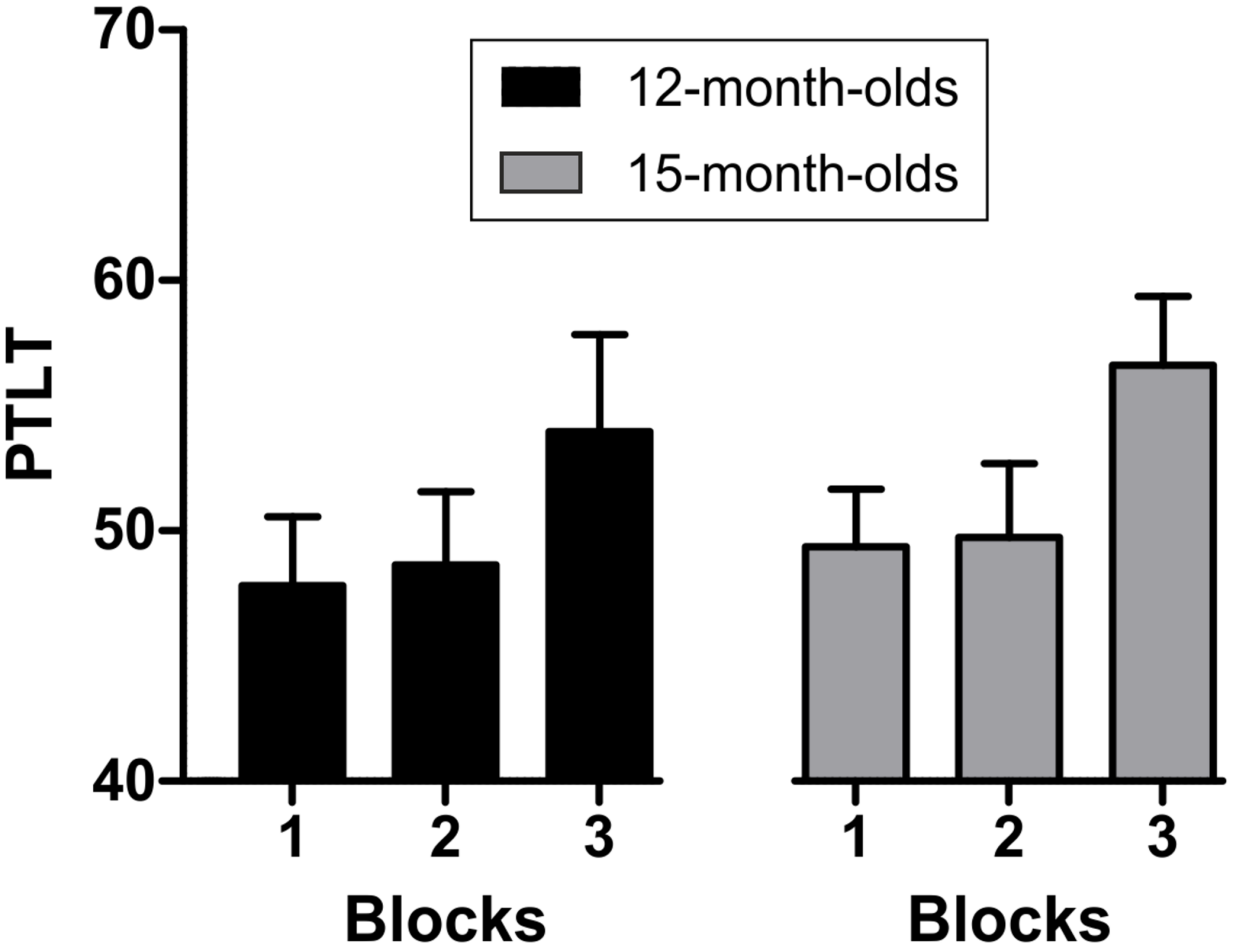
Percentage of total looking time (PTLT) at correct side during the inter-stimulus-interval (ISI) for each age group in each of the three blocks. Bars indicate standard error of the mean.

**Fig 3:**
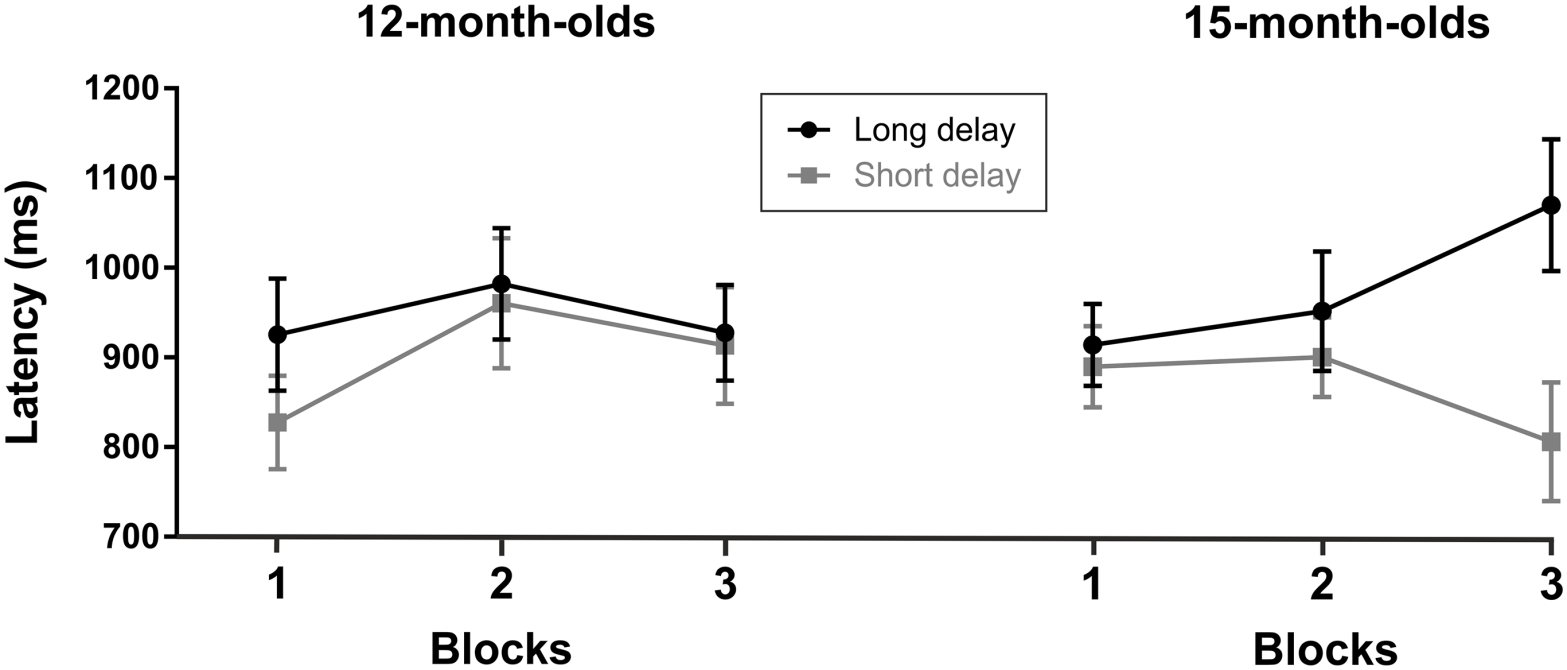
Mean latency (in ms) of first anticipatory look for short and long delays in the first two seconds after the cue in each of the three blocks for each age group. Bars indicate standard error of the mean.
